# Changing epidemiology and survival of HPV-associated oropharyngeal cancer in Denmark: four decades of population-based data

**DOI:** 10.3389/fonc.2026.1982354

**Published:** 2026-10-15

**Authors:** Kathrine Kronberg Jakobsen, Jakob Myllerup Jensen, Joachim Hansen, Christian von Buchwald, Christian Grønhøj

**Affiliations:** Department of Otolaryngology, Head and Neck Surgery & Audiology, Copenhagen University Hospital – Rigshospitalet, Copenhagen, Denmark

**Keywords:** epidemiology, human papillomavirus, incidence, oropharyngeal squamous cell carcinoma, survival

## Abstract

**Background:**

Oropharyngeal squamous cell carcinoma (OPSCC) has undergone substantial epidemiological and clinical changes, with an increasing proportion of human papillomavirus (HPV)-associated tumours and declining tobacco exposure. We investigated temporal changes in patient characteristics, HPV/p16 status, treatment patterns, and survival among patients with OPSCC in Denmark.

**Methods:**

Patients with OPSCC were identified from the Copenhagen Oropharyngeal Cancer Database (2000–2023) and the Danish National Patient Cancer Registry (DCR; 1980–2021). The Copenhagen database provided detailed clinical and pathological data, including HPV/p16 status, while the DCR enabled assessment of long-term survival trends. Temporal changes were evaluated across diagnostic periods. Overall survival, relative survival, and restricted mean survival time were estimated.

**Results:**

Among 3,631 OPSCC patients in the Copenhagen database, median age at diagnosis increased from 59 years in 2000–2005 to 64 years in 2018–2023 (p<0.001). The proportion of HPV+/p16+ patients increased significantly from 43.5% in 2000–2005 to 62.8% in 2018-2023. The proportion of never smokers also increased from 0.5% to 23.5%, while ≥20 pack-years decreased from 73.2% to 52.1% (p<0.001). Treatment patterns changed substantially, with radiotherapy alone decreasing from 87.6% to 27.9% and chemoradiotherapy increasing from 1.6% to 46.3%. Among 9,379 patients in the DCR, five-year relative survival increased from 31.8% in 1980–1984 to 70.1% in 2015-2019. Among HPV+/p16+ patients, five-year overall survival increased from 63.1% in 2000–2005 to 83.7% in 2018–2023.

**Conclusion:**

In Denmark, OPSCC has undergone substantial epidemiological and therapeutic changes accompanied by marked improvements in survival, including among HPV+/p16+ patients.

## Introduction

Oropharyngeal squamous cell carcinoma (OPSCC) has undergone a profound epidemiological and clinical transformation over the past decades. Historically, OPSCC was predominantly associated with tobacco and alcohol exposure and formed part of the broader spectrum of smoking- and alcohol-related head and neck squamous cell carcinomas. However, the increasing recognition of high-risk human papillomavirus (HPV) as a major etiological factor has fundamentally changed the understanding of OPSCC. Over the recent decades, HPV-associated OPSCC has emerged as a distinct disease entity, characterised by different patient demographics, tumour biology, risk-factor profiles, and clinical outcomes compared with HPV-negative disease (1).

The increasing contribution of HPV to OPSCC has been particularly evident in high-income countries, where the incidence of HPV-positive disease has increased alongside a decline in tobacco-related head and neck cancers (2–4). HPV-positive OPSCC is associated with a more favourable response to treatment and substantially better survival than HPV-negative disease, leading to its recognition as a distinct entity in both the American Joint Committee on Cancer (AJCC) and the Union for International Cancer Control (UICC) staging systems (1, 5–7). The changing aetiological landscape has consequently altered not only the prognosis of OPSCC, but also the clinical characteristics of the patients presenting with the disease and the therapeutic considerations surrounding their management.

While numerous studies have described the increasing incidence and prevalence of HPV-associated OPSCC, less is known about how this shift has transformed the overall clinical and prognostic profile of OPSCC at the population level. In particular, temporal changes in patient characteristics, HPV status, disease stage, treatment patterns, and survival may provide important insight into how OPSCC has evolved from a predominantly tobacco- and alcohol-associated malignancy to one increasingly driven by HPV.

The aim of this study was therefore to characterize temporal changes in the clinical characteristics, treatment patterns, and prognosis of patients with OPSCC in parallel with the increasing proportion of HPV-associated disease. Building on previous Danish studies (2, 8) this study specifically adds a focus on long-term survival and aims to quantify survival differences between patients with OPSCC and the Danish general population using matched population controls.

## Method

### Study design

This study was designed as a retrospective registry-based cohort study with an additional matched population-comparator analysis.

### Participants

We included patients diagnosed with oropharyngeal squamous cell carcinoma (OPSCC) in Denmark. Data were obtained from two complementary sources: the *Copenhagen Oropharyngeal Cancer Database* and the *Danish National Patient Cancer Registry* (DCR). The Copenhagen Oropharyngeal Cancer Database contains patients diagnosed with OPSCC within the Eastern Danish healthcare region and covers the period from 2000 to 2023. The database is updated at three-year intervals and covers approximately half of the Danish population.

To extend the study period and identify patients diagnosed prior to establishment of the Copenhagen database, data from the DCR were used for patients diagnosed between 1980 and 2021.

Using data from the Danish Civil Registration System, each patient with OPSCC identified in the DCR was matched to 10 population controls based on age, sex, and calendar year of diagnosis. Controls were selected from the Danish general population and were eligible for matching if they were alive and had not been diagnosed with head and neck cancer before the index date. The index date for each control was defined as the corresponding calendar year of diagnosis of OPSCC in the matched patient. Matching was performed without replacement, such that each control could be assigned to only one patient. Patients for whom 10 eligible controls could not be identified were excluded from the matched analysis. Follow-up for patients and matched controls was calculated from the index date until death, emigration, or the end of the study period, whichever occurred first.

### Data sources

The Copenhagen Oropharyngeal Cancer Database is based on data from the Danish Pathology Registry and the Danish Head and Neck Cancer Group (DAHANCA) database and is supplemented with information extracted from electronic health records (Epic Systems). Patients were identified using their unique Danish personal identification number. Clinical and pathological data were collected in a Research Electronic Data Capture (REDCap) database at Copenhagen University Hospital – Rigshospitalet, Denmark.

Patients with biopsy-confirmed OPSCC diagnosed in Eastern Denmark between January 1, 2000, and December 31, 2023, were identified from the DAHANCA database and the Danish Pathology Data Registry. The DAHANCA database prospectively records clinical characteristics of patients with head and neck cancer, while the Danish Pathology Data Registry is a mandatory nationwide registry containing information on tissue samples collected from patients treated in Denmark. Only the first occurring OPSCC were included in this study. Patients were followed from the date of diagnosis (the date of the first biopsy) until death or the end of the follow-up period. Patients who were alive at the last day of follow-up (1/11-24) were censored on that date.

For patients diagnosed from 2000 onwards, tumour stage was retrospectively classified according to the UICC 8th edition staging system for p16-positive OPSCC. The available UICC 7th edition N categories were converted as follows: N0 remained N0, N1 remained N1, N2a and N2b were recoded as N1, N2c as N2, and N3 remained N3. For T classification, T4a and T4b were combined into T4. The resulting T and N categories were then used to assign UICC 8th edition stage groups according to the following scheme: stage I for T1–2N0–1, stage II for T1–3N2 and T3N0–1, and stage III for T1–3N3 and all T4 tumours.

### p16 testing

p16 expression was assessed by immunohistochemistry (IHC) using the Ventana Benchmark Ultra autostainer and the p16 monoclonal antibody E6H4 with CC1 pretreatment (Roche, Tucson, AZ, USA) throughout the study period. For tumours diagnosed between 2018 and 2023, the OptiView detection kit was used and tumours were considered p16-positive when >70% of tumour cells showed cytoplasmic and/or nuclear staining. Before 2018, the UltraView detection kit was used and the cutoff for p16 positivity was >75% of tumour cells showing cytoplasmic and nuclear staining.

### HPV testing

Formalin-fixed paraffin-embedded (FFPE) tumour tissue was used for HPV DNA extraction and asseessment. Different methods were used for HPV DNA evaluation during the study period. For tumours diagnosed between 2021 and 2023 HPV DNA was detected using the VisionArray HPV Chip 1.0 system (ZytoVision, Bremerhaven, Germany), which detects 41 clinically relevant HPV genotypes. The assay was performed according to the manufacturer’s instructions as previously described (2). Samples with discordant HPV and p16 results were retested. For tumours diagnosed between 2018 and 2020, HPV DNA was detected using either a broad-spectrum GP5+/6+ polymerase chain reaction (PCR) assay or the VisionArray HPV Chip 1.0. For the GP5+/6+ PCR assay, DNA quality was confirmed using a glyceraldehyde-3-phosphate dehydrogenase (GAPDH) control, and samples positive for GP5+/6+ and GAPDH were classified as HPV-positive. The method has previously been described (5). Before 2018, HPV DNA was detected using the GP5+/6+ PCR assay. DNA quality was assessed using GAPDH PCR. HPV genotyping was performed by sequencing of GP5+/6+ amplicons using next-generation sequencing, as previously described (9).

The DCR includes information on cancer diagnoses, including International Classification of Disease 10 revision (ICD-10) codes, MORPHO3 codes, treatment, and survival outcomes. Patients were identified using relevant ICD-10 diagnosis codes and histological information. The included ICD-10 codes were DC01, DC01.9, DC02.4, DC05.1, DC05.2, DC09, DC09.0–DC09.9, DC10.0, DC10.2, DC10.3, DC10.8, and DC10.9. Histologically, cases were classified as squamous cell carcinoma (SCC) and thus included based on the following MORPH3 codes: 80303, 80313, 80323, 80333, 80513, 80523, 80703, 80709, 80713, 80719, 80723, 80733, 80743, 80753, 80763, 80823, 80829, 80833, 80843, 80853, 80863, 81213, 81233, 82303, 82309, and 85603. Patients were included if they were registered with an OPSCC in the DCR between 1/1–1980 and 31/12-2020. Only the first occurring OPSCC were included in the study. Patients were followed from the date of diagnosis until death or the end of the follow-up period. Patients who were alive at the last day of follow-up (1/1-24) were censored on that date.

The two data sources served complementary purposes: the DCR enabled analyses covering a longer historical period, whereas the Copenhagen Oropharyngeal Cancer Database provided more detailed clinical and pathological data, including HPV and p16 status. The specific data source used for each analysis is indicated in the corresponding tables and figures.

### Study size

From the Copenhagen Oropharyngeal Cancer Database, a total of 3,631 patients were included.

A total of 9,057 patients with OPSCC were identified in the DCR. One patient was lost to follow-up and was excluded from the analysis, resulting in a total of 9,056 patients. Eighteen patients emigrated from Denmark during follow-up and were censored at the date of emigration.

For the matched analysis, each patient was matched with 10 population controls based on age, sex, and year of diagnosis. Seven patients could not be assigned eligible controls and were therefore excluded from the matched analysis, leaving 9,049 patients for the matched analysis. Of these, eight controls were excluded because they died before the matching date. In total, 90,482 controls were included in the matched analysis.

### Study outcomes

The primary objective was to characterise temporal changes in the clinical characteristics of patients with OPSCC in parallel with the increasing proportion of HPV-associated disease. The primary outcomes were temporal changes in HPV/p16 status, patient characteristics, tumour stage, and treatment modality. Secondary outcomes included temporal changes in relative survival, overall survival, long-term survival, and survival compared with matched population controls.

For temporal analyses, the study periods were divided into approximately equal intervals. For data from the Copenhagen database, the study period was divided into four periods: 2000–2005, 2006–2011, 2012–2017, and 2018–2023. For data from the DCR, the study period was divided into nine periods: 1980–1984, 1985–1989, 1990–1994, 1995–1999, 2000–2004, 2005–2009, 2010–2014, 2015–2019, and 2020–2021 thereby extending the analysis to include the full available study period.

### Statistical analysis

Descriptive statistics were used to summarize the study population and tumour characteristics. Categorical variables were presented as numbers and percentages, while continuous variables were summarized using appropriate measures of central tendency and dispersion.

### Overall survival

Overall survival was estimated using the Kaplan–Meier method, with time calculated from the date of OPSCC diagnosis to the date of death or last available follow-up. Patients who were alive at the end of follow-up were censored at their last known date of follow-up. Kaplan–Meier curves were generated to illustrate overall survival across diagnostic periods, and differences between survival curves were assessed using the log-rank test.

### Long-term survival

To assess changes in long-term survival, patients were grouped according to diagnostic period, and Kaplan–Meier estimates of overall survival at 5, 10, and 15 years were calculated. For each survival interval, only patients with sufficient potential follow-up were included. Given follow-up through 2024 (last day of follow-up 1 January 2024), patients diagnosed after 2018, 2013, and 2008 were excluded from the 5-, 10-, and 15-year analyses, respectively. Overall survival at each time point was estimated using the Kaplan–Meier method, with 95% confidence intervals.

### Relative survival

Relative survival was estimated using data from the DCR to assess survival among patients with OPSCC relative to the expected survival in the general Danish population. Expected survival was derived from Danish population life tables obtained from Statistics Denmark and stratified by age, sex, and calendar year. The relative survival rate was calculated as the ratio of observed to expected survival in Denmark, adjusted for age, sex, and calendar year, using the R package ‘relsurv’. Relative survival curves were estimated using the Ederer II method, with expected survival determined from the population life tables according to age, sex, and calendar year.

### Survival compared with matched controls

Restricted mean survival time (RMST) was used to quantify differences in survival between patients with OPSCC and matched controls over predefined follow-up periods. This measure represents the expected time alive within a specified follow-up horizon (10). Estimates were obtained at 5, 10, and 15 years after diagnosis for both groups, and the between-group difference in RMST was calculated with corresponding 95% confidence intervals. Thus, the difference in RMST represents the difference in accumulated survival time between patients with OPSCC and their matched controls within each specified time horizon.

All statistical analyses were performed using RStudio (version 2025.05.1). Statistical significance was defined as a two-sided P value ≤0.05.

### Results

A total of 3,631 patients with OPSCC were included from the Copenhagen database. The median age at diagnosis increased significantly over time, from 59 years (IQR, 52.8–66.0) in 2000–2005 to 64 years (IQR, 57–71) in 2018–2023 (p<0.001). The sex distribution remained stable, with men accounting for approximately 71–74% of patients across all periods (p=0.6) (**Table 1**). A marked shift in HPV/p16 status was observed over time (p<0.001). The proportion of HPV+/p16+ tumours increased from 43.5% in 2000–2005 to 62.8% in 2018–2023, while the proportion of HPV−/p16− tumours decreased from 40.7% to 24.8%. The proportions of both HPV−/p16+ and HPV+/p16− tumours also decreased over time. The distribution of HPV/p16 status varied by age and diagnosis period (**Figure 1**). Across all age groups, HPV+/p16+ tumors increased over time, while HPV−/p16− tumors decreased. The increase was most pronounced among patients aged <50 years, with HPV+/p16+ tumors rising from approximately 50% in 2000–2005 to 90% in 2018–2023, compared with an increase from 30% to 60% among patients aged ≥70 years.

**Table 1:** Baseline characteristics of patients based on data from the Copenhagen database.

Variable	Category	Overall	2000-2005	2006-2011	2012-2017	2018-2023	p
**n**		3631	428	568	1229	1405	
**Age**		62.00 [55.00, 69.00]	59.00 [52.75, 66.00]	59.00 [53.00, 65.00]	62.00 [55.00, 69.00]	64.00 [57.00, 71.00]	<0.001
**Sex**	Female	991 (27.3)	126 (29.4)	148 (26.1)	342 (27.8)	374 (26.6)	0.6
	Male	2640 (72.7)	302 (70.6)	420 (73.9)	887 (72.2)	1031 (73.4)	
**HPV/p16 status (%)**	HPV-/p16-	1105 (30.4)	174 (40.7)	184 (32.4)	398 (32.4)	348 (24.8)	<0.001
	HPV+/p16+	2088 (57.5)	186 (43.5)	313 (55.1)	706 (57.4)	883 (62.8)	
	HPV-/p16+	151 (4.2)	31 (7.2)	38 (6.7)	54 (4.4)	28 (2.0)	
	HPV+/p16-	134 (3.7)	37 (8.6)	21 (3.7)	32 (2.6)	44 (3.1)	
	Unknown	153 (4.2)	0 (0.0)	12 (2.1)	39 (3.2)	102 (7.3)	
**Smoking status (%)**	Never smoker	353 (9.7)	2 (0.5)	1 (0.2)	26 (2.1)	324 (23.1)	<0.001
	< 10 pack years	219 (6.0)	19 (4.4)	44 (7.7)	72 (5.9)	84 (6.0)	
	>=10 or < 20 pack years	333 (9.2)	37 (8.6)	55 (9.7)	95 (7.7)	145 (10.3)	
	>=20 pack years	1912 (52.7)	267 (62.4)	305 (53.7)	614 (50.0)	726 (51.7)	
	Unknown	814 (22.4)	103 (24.1)	163 (28.7)	422 (34.3)	126 (9.0)	
**Alcohol status (%)**	No abuse	1664 (45.9)	0 (0.0)	63 (11.1)	655 (53.3)	945 (67.6)	<0.001
	Previous abuse	274 (7.6)	0 (0.0)	8 (1.4)	109 (8.9)	157 (11.2)	
	Current abuse	226 (6.2)	0 (0.0)	4 (0.7)	89 (7.2)	133 (9.5)	
	Current heavy abuse	297 (8.2)	0 (0.0)	5 (0.9)	140 (11.4)	152 (10.9)	
	Unknown	1170 (32.2)	428 (100.0)	488 (85.9)	236 (19.2)	18 (1.3)	
**UICC 8 stage (%)**	1	1483 (40.9)	152 (35.5)	235 (41.5)	523 (42.6)	573 (40.8)	0.002
	2	677 (18.7)	74 (17.3)	102 (18.0)	230 (18.7)	271 (19.3)	
	3	601 (16.6)	87 (20.3)	86 (15.2)	178 (14.5)	250 (17.8)	
	4	766 (21.1)	107 (25.0)	120 (21.2)	252 (20.5)	286 (20.4)	
	Unknown	104 (2.9)	8 (1.9)	25 (4.4)	46 (3.7)	25 (1.8)	
**Treatment type (%)**	No treatment	131 (3.6)	20 (4.7)	13 (2.3)	36 (2.9)	62 (4.4)	<0.001
	Radiotherapy	1360 (37.5)	375 (87.6)	240 (42.3)	353 (28.7)	391 (27.9)	
	Chemo+radiotherapy	1554 (42.8)	7 (1.6)	287 (50.5)	612 (49.8)	648 (46.3)	
	Surgery	294 (8.1)	2 (0.5)	1 (0.2)	132 (10.7)	159 (11.3)	
	Surgery and radiotherapy	28 (0.8)	0 (0.0)	0 (0.0)	14 (1.1)	14 (1.0)	
	Surgery and chemo-radiotherapy	25 (0.7)	0 (0.0)	0 (0.0)	11 (0.9)	14 (1.0)	
	chemotherapy	1 (0.0)	0 (0.0)	0 (0.0)	1 (0.1)	0 (0.0)	
	Palliative treatment	217 (6.0)	18 (4.2)	21 (3.7)	65 (5.3)	113 (8.1)	
	Unknown	21 (0.6)	6 (1.4)	6 (1.1)	5 (0.4)	4 (0.3)	

Bold values indicate overall characteristics for the study population.

**Figure 1:**
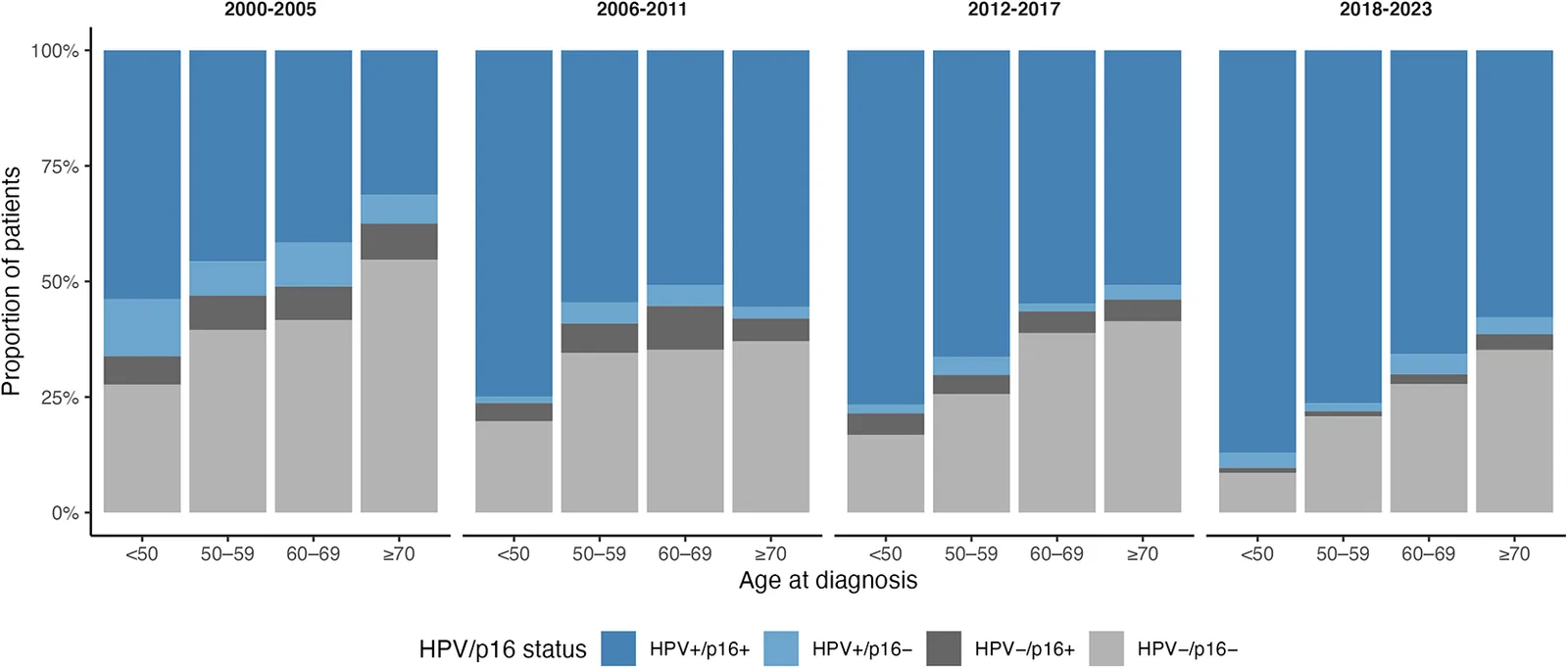
Distribution of HPV/p16 status by age group and diagnosis period.

Smoking patterns changed substantially during the study period (p<0.001). The proportion of never smokers increased from 0.5% in 2000–2005 to 23.1% in 2018–2023, whereas the proportion of patients with ≥20 pack-years decreased from 62.4% to 51.7%. Alcohol status was unavailable for all patients in 2000–2005 and for 85.9% in 2006–2011 and interpretation is thus not possible.

The distribution of UICC stage was broadly similar across the study periods, with stage I being the most common stage throughout (35.5–42.6%). Treatment patterns, however, changed substantially over time (p<0.001). Radiotherapy alone was the predominant treatment modality in 2000–2005, accounting for 87.6% of patients, but decreased to 27.9% in 2018–2023. In contrast, combined chemoradiotherapy increased from 1.6% to 46.3%, while surgery increased from 0.5% to 11.3%. The proportion receiving palliative treatment also increased over time, from 4.2% to 8.1%.

### Survival

Relative survival improved across the diagnostic periods based on data from the DCR. The analysis includes a total of 9,056 patients. Five-year relative survival increased from 31.0% (95% confidence interval (CI), 25.5–37.5%) for patients diagnosed in 1980–1984, compared with 70.3% (95% CI, 68.1–72.6%) in 2015-2019 (**Figure 2**).

**Figure 2:**
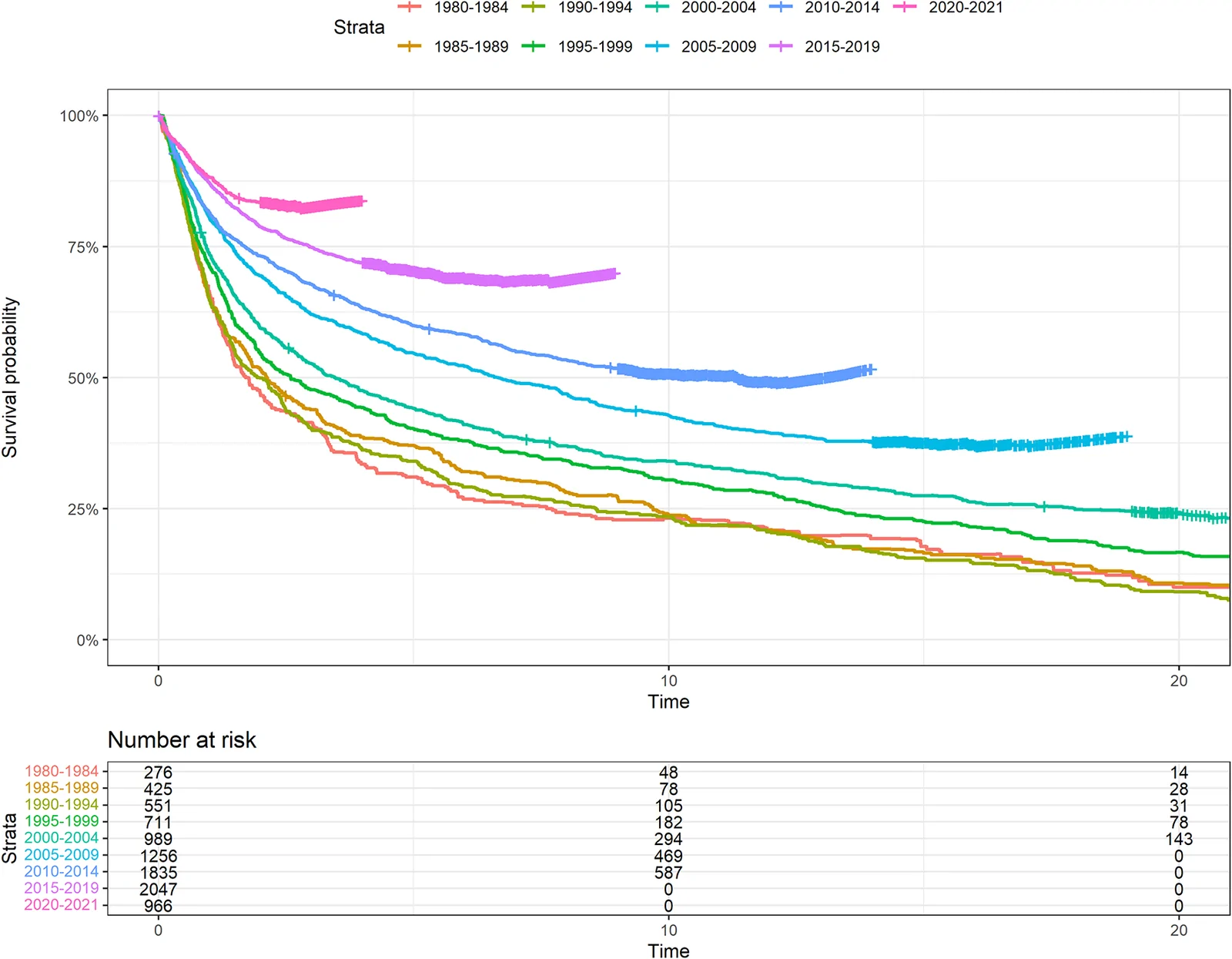
Relative survival stratified on time period based on data from the Danish national patient registry.

The proportion of long-term survivors increased across the diagnostic periods (**Figures 3A, B**), from a proportion of 27.2% among patients diagnosed in 1980–1984 surviving at least 5 years to 64.4% among those diagnosed in 2015–2019. Similarly, the proportion surviving at least 10 years increased from 17.4% in the 1980–1984 cohort to 40.5% among patients diagnosed in 2010–2014. The proportion of patients surviving at least 15 years also increased over time, from 10.9% among patients diagnosed in 1980–1984 to 26.7% among those diagnosed in 2000–2008. Estimates of 10- and 15-year survival were not available for the most recent diagnostic periods because these patients had not accumulated sufficient follow-up time.

**Figure 3:**
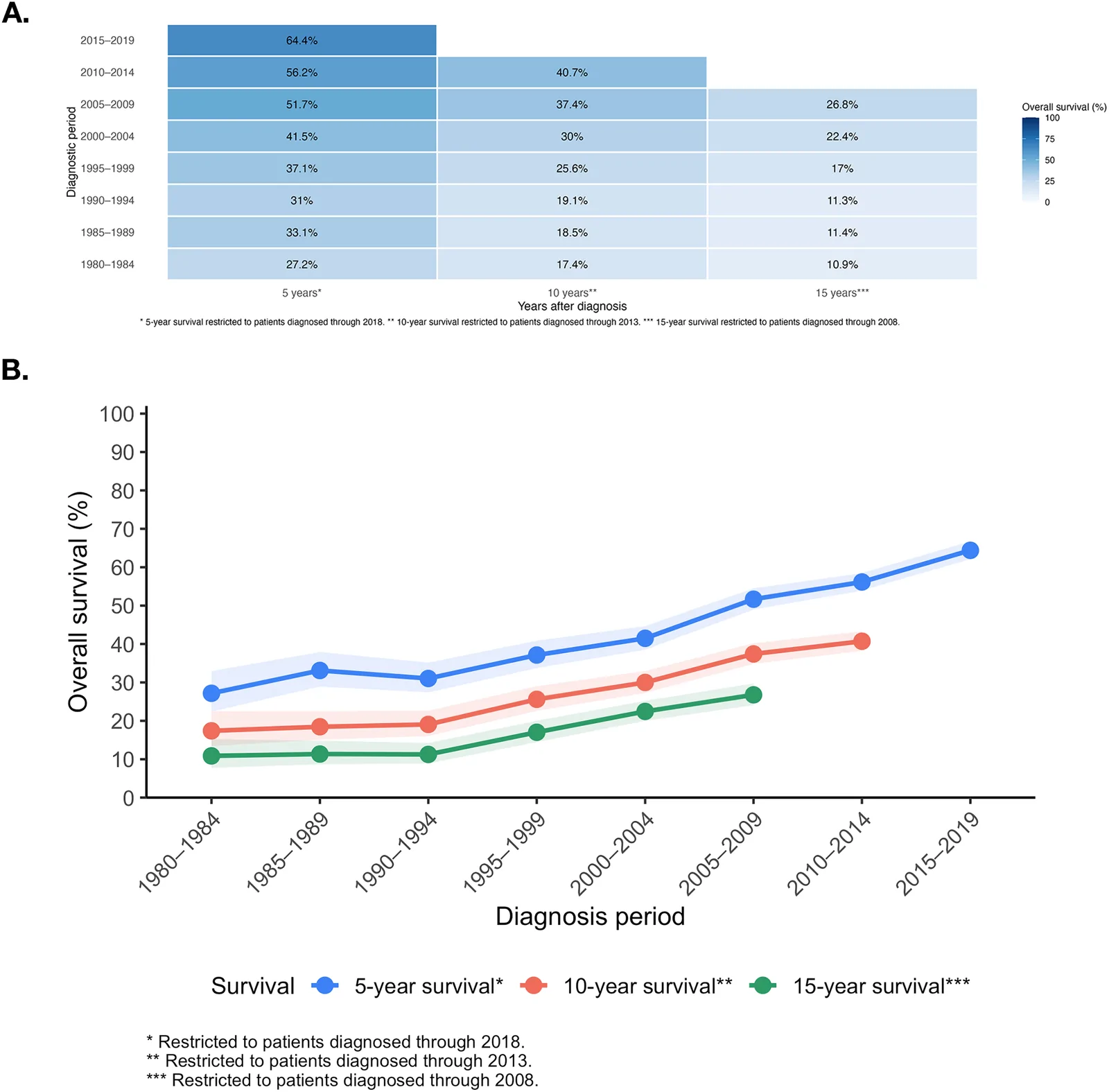
**(A)** and **(B)** Kaplan–Meier estimates of 5-, 10-, and 15-year overall survival among patients with OPSCC across diagnostic periods.

The analysis of RMST demonstrated a progressively larger difference in accumulated survival between patients with OPSCC and matched controls with increasing follow-up (**Table 2**). Within the first 5 years after diagnosis, RMST was 3.40 years (95% CI, 3.36–3.44) among patients with OPSCC compared with 4.77 years (95% CI, 4.76–4.77) among controls, corresponding to a difference of −1.37 years (95% CI, −1.41 to −1.33; P<0.001). At 10 years, RMST was 5.69 years (95% CI, 5.60–5.77) and 9.04 years (95% CI, 9.02–9.05), respectively, corresponding to a difference of −3.35 years (95% CI, −3.44 to −3.26; P<0.001). At 15 years, RMST was 7.37 years (95% CI, 7.24–7.50) among cases and 12.76 years (95% CI, 12.73–12.79) among controls, corresponding to a difference of −5.39 years (95% CI, −5.52 to −5.25; p<0.001).

**Table 2:** Restricted mean survival time for patients with OPSCC and matched controls.

Follow-up	OPSCC (years)	Matched controls (years)	Difference in RMST (years)	p-value
5 years	3.40 (3.36–3.44)	4.77 (4.76–4.77)	−1.37 (−1.41 to −1.33)	<0.001
10 years	5.69 (5.60–5.77)	9.04 (9.02–9.05)	−3.35 (−3.44 to −3.26)	<0.001
15 years	7.37 (7.24–7.50)	12.76 (12.73–12.79)	−5.39 (−5.52 to −5.25)	<0.001

When stratified on HPV+/p16+ patients we observed a similar improvement in survival based on data from the Copenhagen database (**Figure 4**). Five-year overall survival increased from 63.1% (95% CI, 57.1–69.8%) among patients diagnosed in 2000–2005 to 76.3% (95% CI, 71.9–80.9%) in 2006–2011, remained at 76.3% (95% CI, 73.0–79.9%) in 2012–2017, and increased further to 83.7% (95% CI, 80.5–87.0%) in 2018–2023. Thus, five-year overall survival among HPV+/p16+ patients increased by 20.6 percentage points between the earliest and latest diagnostic periods.

**Figure 4:**
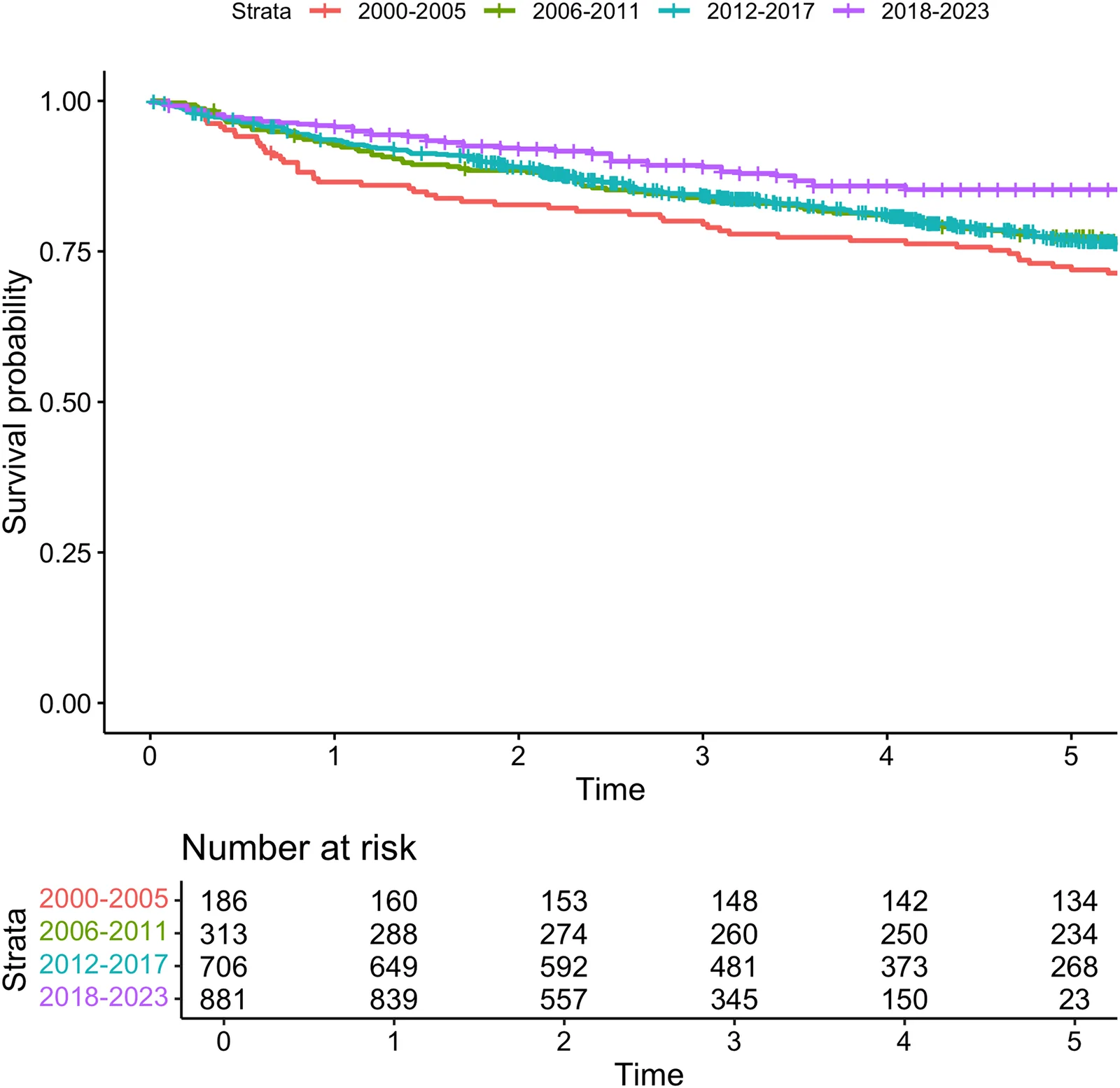
Overall survival stratified on time period for HPV+/p16+ patients based on data from the Copenhagen oropharyngeal cancer database.

## Discussion

In this study, we observed substantial changes in the epidemiology, treatment, and survival of patients with OPSCC over time.

Treatment patterns changed considerably during the study period, with radiotherapy alone becoming less common and combined treatment modality increasing. These changes may reflect advances in the multidisciplinary management of OPSCC, including centralisation of treatment decisions in multidisciplinary tumour boards, refinement of positron emission tomography/computed tomography (PET/CT)-based staging, and the introduction of transoral robotic surgery as a treatment option for selected early-stage tumours (11), although this study did not directly evaluate the drivers of these treatment shifts. Notably, despite the favourable prognosis of HPV+/p16+ OPSCC, treatment de-intensification has not been established as standard practice. Randomised trials evaluating reduced-intensity treatment in this subgroup, including the De-ESCALaTE HPV and Radiation Therapy Oncology Group (RTOG) 1016 trials, failed to demonstrate non-inferiority of cetuximab-based regimens compared with cisplatin-based chemoradiotherapy, and de-escalation therefore remains investigational inside clinical trials (12). This is consistent with continued reliance on combined-modality treatment, rather than de-escalated single-modality regimens, despite the growing proportion of HPV+/p16+ tumours observed in our cohort.

Survival improved substantially over the study period. In the DCR cohort, five-year relative survival increased from 31.9% in patients diagnosed in 1980–1984 to 70.1% in those diagnosed in 2015–2019. A similar improvement was observed among HPV+/p16+ patients based on data from the Copenhagen Database, for whom five-year overall survival increased from 63.1% in 2000–2005 to 83.7% in 2018–2023. This improvement is consistent with the established prognostic significance of HPV status first demonstrated by Ang et al. (13), and extends findings from previous Danish nationwide studies reporting improving OPSCC survival over time (5, 8). Importantly, survival also improved within the HPV+/p16+ subgroup, indicating that the increasing proportion of HPV-driven tumours alone does not fully account for the observed population-level improvement in survival. Possible explanations for the remaining improvement include refinements in staging accuracy, changes in patient selection, and improvements in treatment delivery over time. The introduction of HPV-specific staging in the eighth edition of the AJCC/UICC TNM classification during the study period should be considered when interpreting temporal comparisons of stage-stratified outcomes. However, as patients diagnosed from 2000 onwards were retrospectively classified according to UICC 8 using the available UICC 7 T and N categories, differences in stage distribution cannot be attributed solely to the change in staging system. The observed temporal changes may reflect underlying differences in tumour biology and disease presentation, including the increasing proportion of HPV/p16-positive OPSCC. Concurrently, declining tobacco exposure in the general population is likely to have contributed independently to the changing tumour biology (2, 14, 15).

The present study builds on previous Danish studies of OPSCC, including the Copenhagen cohort reported by Michaelsen et al. (2) and the nationwide Danish Cancer Registry study by Jakobsen et al (8). While there is substantial cohort overlap, the present study extends these findings by providing a broader longitudinal assessment of clinical characteristics, tumour stage, and treatment patterns, with particular emphasis on long-term survival. The increasing proportion of long-term survivors further demonstrates the substantial improvement in prognosis. However, the comparison with matched population controls showed that patients with OPSCC continued to experience a substantial survival deficit, which increased with longer follow-up. This finding highlighting that improved cancer survival does not necessarily translate into survival comparable to that of the general population. These findings emphasise the growing population of long-term OPSCC survivors and the increasing importance of survivorship and management of late treatment-related effects, including dysphagia, xerostomia, and other functional sequelae of chemoradiotherapy, which can persist for years after treatment completion and substantially affect quality of life (16). This underscores the need for structured, long-term survivorship programmes as an increasing share of the OPSCC population moves from active treatment into extended follow-up.

A key strength of this study is the use of two complementary data sources. The evaluation of survival trends over more than four decades was enabled by the DCR, while the Copenhagen database provided detailed clinical and pathological information, including HPV/p16 status. Limitations include limited availability of HPV/p16 data in the DCR, and shorter follow-up in the most recent cohorts. Additionally, changes in staging classification during the study period may limit direct comparability of stage-specific outcomes across diagnostic periods. Further, HPV DNA and p16 status were assessed using different methods and protocols over the study period, including different HPV DNA assays and p16 detection kits and positivity thresholds, which may have introduced some degree of methodological heterogeneity in the classification of HPV/p16 status. Lastly, the completeness of smoking data varied over the study period, and differences in the ascertainment or recording of smoking status over time may have contributed to the observed temporal increase in the proportion of never-smokers. Therefore, this finding should be interpreted with consideration of potential temporal ascertainment bias.

In conclusion, OPSCC in Denmark has undergone substantial epidemiological and therapeutic changes, accompanied by marked improvements in both short- and long-term survival. These improvements were also evident among HPV+/p16+ patients, underscoring continued progress in the management of OPSCC. Importantly, the present study demonstrates that these improvements extend to long-term survival and provides insight into survival relative to the Danish general population.

## Data Availability

The datasets presented in this article are not readily available because the individual-level registry data used in this study are not publicly available and cannot be shared by the authors due to Danish data protection legislation and legal restrictions governing access to registry-based health data. The data are held by the relevant Danish data authorities. Researchers wishing to access the underlying registry data must apply directly to the relevant data authorities and fulfill the applicable legal, ethical, and data protection requirements for an independent research project. Requests to access the datasets should be directed to Kathrine Kronberg Jakobsen, kathrine.kronberg.jakobsen@regionh.dk.
